# Impact of Organism Reporting from Endotracheal Aspirate Cultures on Antimicrobial Prescribing Practices in Mechanically Ventilated Pediatric Patients

**DOI:** 10.1128/jcm.00930-22

**Published:** 2022-10-11

**Authors:** Andrea M. Prinzi, Rachel L. Wattier, Donna J. Curtis, Sonja I. Ziniel, Allyson Fitzgerald, Kelly Pearce, Sarah K. Parker

**Affiliations:** a Clinical Science, University of Colorado Anschutz Medical Campus, Aurora, Colorado, USA; b Department of Pediatrics, University of Colorado School of Medicine, Aurora, Colorado, USA; c Department of Pathology, Microbiology, Children’s Hospital Colorado, Aurora, Colorado, USA; d Department of Pediatrics, University of California–San Francisco, San Francisco, California, USA; University of Iowa College of Medicine

**Keywords:** microbiology, culture, diagnostics, laboratory, stewardship

## Abstract

Endotracheal aspirate cultures (EACs) help diagnose lower respiratory tract infections in mechanically ventilated patients but are limited by contamination with normal microbiota and variation in laboratory reporting. Increased use of EACs is associated with increased antimicrobial prescribing, but the impact of microbiology reporting on prescribing practices is unclear. This study was a retrospective analysis of EACs from mechanically ventilated patients at Children’s Hospital Colorado (CHCO) admitted between 1 January 2019 and 31 December 2019. Chart review was performed to collect all culture and Gram stain components, as well as antibiotic use directed to organisms in culture. Reporting concordance was determined for each organism using American Society for Microbiology guidelines. Days of therapy were calculated for overreported and guideline-concordant organisms. A multivariable model was used to assess the relationship between organism reporting and total days of therapy. Overall, 448 patients with 827 EACs were included in this study. Among patients with tracheostomy, 25 (8%) organisms reported from EACs were overreported and contributed 48 days of excess therapy, while 227 (29%) organisms from the EACs of endotracheally intubated patients were overreported, contributing 472 excess days of therapy. After adjustment, organism overreporting was associated with a >2-fold-higher rate of antimicrobial therapy than guideline-concordant reporting (incident rate ratio [IRR], 2.83; 95% confidence interval [CI], 1.23, 6.53; *P* < 0.05). Overreported organisms from respiratory cultures contribute to excess antimicrobial therapy exposure in mechanically ventilated patients. Microbiology laboratories have an opportunity to mitigate antimicrobial overuse through standardized reporting practices.

## INTRODUCTION

Endotracheal aspirate cultures (EACs) are commonly obtained from mechanically ventilated pediatric patients (1). A positive culture alone is not diagnostic of lower respiratory tract infection (LRTI) due to contamination with normal oropharyngeal microbiota, frequent respiratory tract colonization in mechanically ventilated patients, and poor correlation of EAC results with clinical LRTI (2–4). Studies have demonstrated that increased EAC use may potentiate excessive antimicrobial use (5, 6). Although the American Society for Microbiology (ASM) provides guidelines for reporting primary pathogens from lower respiratory specimens (7), reporting practices vary (8), and laboratories may encounter implementation barriers, such as pressure from frontline clinicians to report additional organisms. Furthermore, the impact of reporting organisms besides primary pathogens is not well known.

Clinically focused diagnostic interventions for EACs can reduce their use without negatively impacting clinical outcomes (9). However, the recent VAIN2 study demonstrated that even with implementation of a clinical algorithm for EACs, nearly 73% of patients received unnecessary therapy due to clinicians’ tendency to treat “positive” EACs regardless of treatment guidelines or recommendations (10). Recent stewardship study data suggest that adding nudging comments to respiratory culture reports may impact prescribing practices (11), but the impact of organism reporting from EACs has not been demonstrated. This study aimed to evaluate the impact of EAC overreporting by the microbiology laboratory on the clinician’s prescribing choices.

## MATERIALS AND METHODS

This was a retrospective cohort study at a 444-bed academic pediatric hospital. All patients admitted between 1 January 2019 and 31 December 2019 who received an EAC were assessed for eligibility. Patients were excluded if they had cystic fibrosis, were admitted to pediatric oncology or bone marrow transplant services, or were admitted for less than 3 days (*n* = 75) (see the flowchart labeled Supplement 1 in the supplemental material). Cultures with no growth, missing data, duplicated, or canceled in the laboratory were excluded (*n* = 207). Repeat cultures growing the same organism and that resulted during the same antibiotic treatment period were excluded (*n* = 57). Only data from a patient’s first hospitalization in 2019 were included. This study was classified as exempt research (secondary research not requiring consent) per the University of Colorado Institutional Review Board.

### Patient data.

REDcap was used for all data collection (12). Demographics and medical conditions at admission were collected for each participant from the medical record. The airway device at the time of culture collection was documented (i.e., tracheostomy or endotracheal tube [ETT]). Notes on patient history taken at the time of admission were reviewed by an infectious disease physician (D.J.C.) and a clinical microbiologist (A.M.P.). Patients with at least one existing condition present prior to admission and documented in the admission notes were classified as having one or more underlying conditions. (Table 1).

**TABLE 1:** Characteristics of patients with endotracheal tube and tracheostomy

Characteristic	Result for patients (*n* = 448)^*a*^
Age	
Median (IQR)	10 mo (1 mo–7 yrs)
0–27 days	65 (15)
28 days–12 mo	121 (27)
13 mo–2 yrs	77 (17)
3–5 yrs	47 (10)
6–11 yrs	66 (15)
12–18 yrs	57 (13)
>18 yrs	15 (3)
Gender	
Female	188 (42)
Male	256 (57)
Unknown	4 (1)
Race/ethnicity	
American Indian/Alaska Native	12 (3)
Asian	8 (2)
Black, not Hispanic or Latino	43 (10)
Hispanic or Latino	112 (25)
More than 1 race, Hispanic or Latino	23 (5)
More than 1 race, not Hispanic or Latino	14 (3)
Other	12 (2)
Unknown/not specified	29 (6)
White, not Hispanic or Latino	195 (44)
Length of stay (days)	
Median, IQR	15 (7–55)
Minimum	3
Maximum	943
Cumulative ventilator days	
Median, IQR	3 (2–7)
Min	<24 h
Max	66
≥1 underlying condition at admission	282 (63)
≥2 conditions^*b*^	126 (45)
Congenital heart condition	40 (14)
Prematurity	35 (12)
Other (i.e., Dravet syndrome, DiGeorge syndrome, solid organ transplant)	26 (9)
Lung disease	19 (7)
Cerebral Palsy	11 (4)
Tumor	9 (3)
Trisomy 21	7 (2)
Diaphragmatic hernia	5 (1)
Metabolic disorder	4 (1)

a All values are shown as number (percentage) unless noted as median (IQR).

b Includes multiple conditions (i.e., “chronic lung disease, trach/vent dependent, g-tube dependent”) and patients with multiple congenital anomalies.

### EAC data.

**FIG 1:**
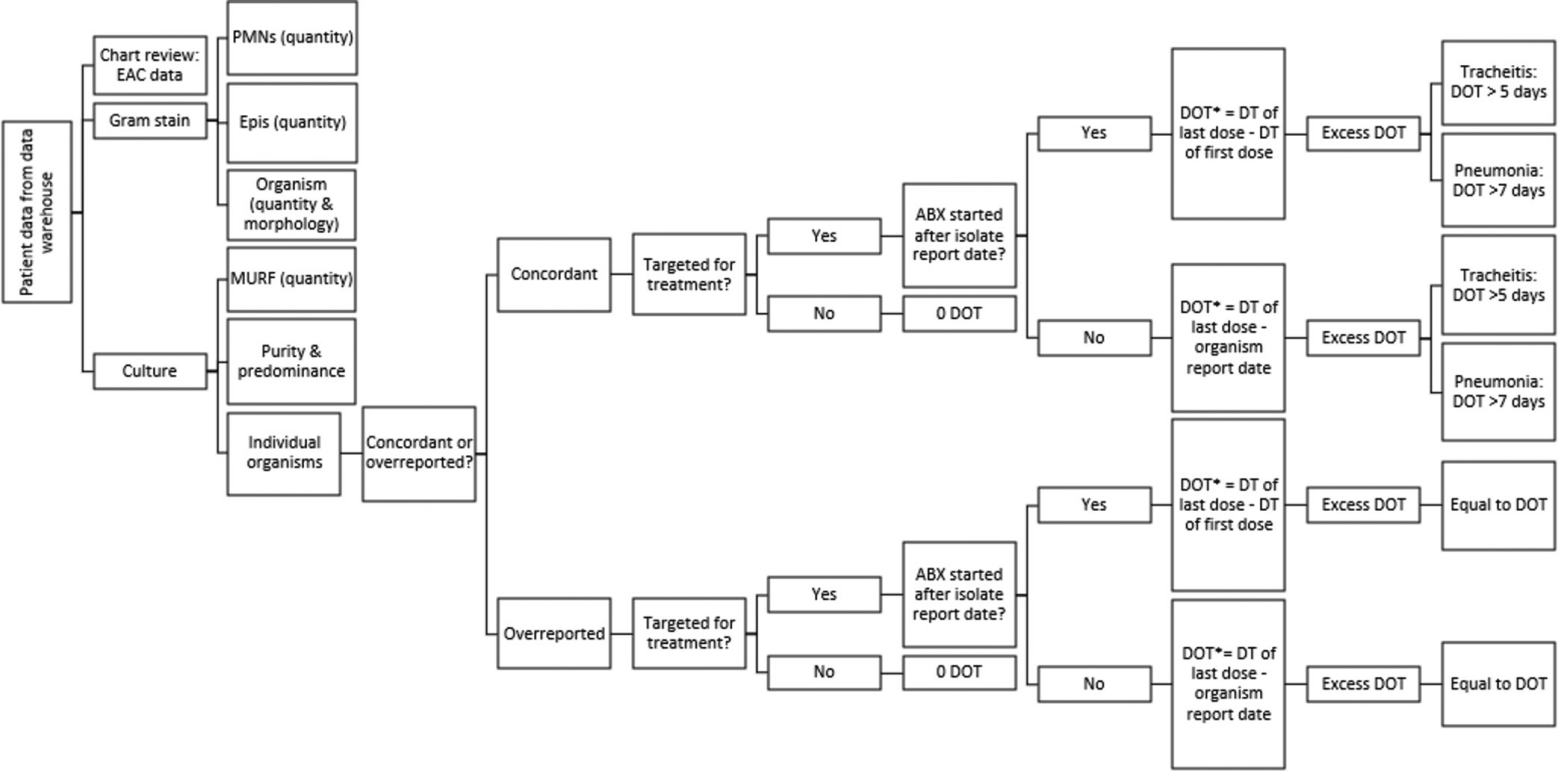
Flowchart of methods. DT, date and time; DOT, days of therapy; ABX, antibiotic. “DOT*” indicates that the total DOT includes empirical therapy used before the organism report and is the difference between the antibiotics’ start dates and end dates. Postreporting DOT accounts only for the DOT that occurred after an organism was reported and is the difference between the organism report date(s) and antibiotic end date(s).

All patient charts were reviewed for culture data for each EAC sent during the included admission. Quantities (rare, few, moderate, or heavy) and morphologies of cells and organisms were documented for Gram stain (GS) and culture (Fig. 1). In the present study’s hospital laboratory, a Gram stain is made from the endotracheal aspirate (EA) specimen when it is received and the results are reported the same day. Microbiologists report the quantity and morphology of all organisms visualized and the quantity of epithelial and polymorphonuclear cells (PMNs). After the specimen has been plated and incubated for 18 to 24 h, plates are examined for growth. Three or more commensal organisms without a predominant organism are reported as mixed upper respiratory flora (MURF) in the patient’s chart, which aligns with the ASM guidelines for reporting from lower respiratory tract specimens (7). For this study, the quantity of MURF reported was recorded, and results were categorized as “MURF only” if “MURF” was reported without individually identified organisms. Predominant organisms may be selected for identification and susceptibility testing dependent on patient history, organism type, and microbiologist discretion. Individual isolates were defined as organisms that were identified and reported from culture (Fig. 1). Data for up to three individually reported organisms were collected for each culture, with date and time when each was first reported from culture. Culture results were reviewed by a clinical microbiologist (A.M.P.) to determine if the culture had pure growth of a single organism or if predominant organisms were reported. Predominant organisms were defined as up to two organisms growing in greater quantities than other organisms in culture.

### Organism reporting concordance.

The ASM guidelines for reporting primary pathogens from lower respiratory specimens (7) were used to determine the concordance of each organism reported from culture (Fig. 1). A culture adjudication process was performed by two clinical microbiologists (A.M.P. and A.F.) to ensure the consistency of organism classification. At the time of adjudication, both microbiologists were blind to clinical information or antibiotic choice. Only a summary of the microbiology report containing Gram stain and culture results was available for review. Twenty cultures were randomly selected and independently reviewed and resulted in 100% agreement between the microbiologists. The remaining organism assignments were made by one clinical microbiologist (A.M.P.). All organisms from EACs were reviewed for concordance with ASM guidelines (Fig. 1), with classification as “concordant” if guidelines recommend reporting or “overreported” if guidelines do not recommend reporting (see the flowcharts and figure labeled Supplement 3 in the supplemental material). In clinical microbiology, a report of “MURF only” means that a predominant pathogen was not present. Full identification and reporting of colonizing organisms that are not predominant are considered poor laboratory practice. A culture result of “MURF only” suggests that the microbiologists are following recommended reporting practices by not identifying and reporting nonpredominant colonizing flora (Supplement 3). Due to this, MURF was always considered to be concordant. Only organism data initially reported by the microbiology laboratory were recorded. If a clinician called and requested a complete identification of an organism, this was documented, but only the identification initially issued by the microbiology laboratory was used for analysis.

### Antibiotic association with EACs.

Each patient’s chart was reviewed for initiation of an antibiotic with activity against the reported organism within 24 to 48 h before or after each organism was reported. Provider notes were reviewed for language detailing the reason for starting an antibiotic. If the notes stated that an organism growing from the EAC was specifically treated, that organism was considered “targeted” for treatment and antibiotic information was collected (e.g., “treating for Staphylococcus aureus tracheitis”). If the reason for antibiotic initiation was unclear in the provider notes, the antimicrobial stewardship dashboard in Epic was used to determine antibiotic indication for each organism. The dashboard provides data on antibiotic, dose, start and end dates, and indications for use entered at the time of order entry. For example, if an antibiotic used to target the organism of interest was initiated within 24 h of the organism report date from culture and had an indication of either “tracheitis,” “pneumonia/sinusitis,” or specific mention of the organism growing from the EAC (i.e., “Stenotrophomonas maltophilia in EAC”), the organism was considered targeted for treatment and the dates and times of the first and last dose were collected. For nearly all cultures, three antibiotics were the maximum number prescribed to treat a given organism, and data for up to three antibiotics were collected for every organism reported from each EAC in the data set (Fig. 1).

### Antibiotic days of therapy.

Antibiotic days of therapy (DOT) were calculated by determining the difference between the end date and time of therapy and the start date and time of therapy for each organism. “Postreporting DOT” was calculated in two ways: if therapy was started after an organism was reported, then all DOT were collected for that antibiotic. If an antibiotic was started before the organism was reported (empirical therapy) but continued after the organism report date, the DOT occurring after the organism report date were collected. This calculation was performed by finding the difference between the end date and time of therapy and the date and time that the organism was reported. The reasoning for this methodology was to effectively assess the prescribing choices driven by microbiology laboratory reporting. To reduce to risk of misclassification bias, total DOT (including empirical therapy) were also collected and calculated from the difference between the start and end dates of therapy regardless of the organism report date and time. If an organism was targeted with multiple antibiotics, the DOT of each antibiotic were added together (Fig. 1).

Excess DOT were calculated differently between the concordant organism group and the overreported organism group (Fig. 1). In the concordant group, excess DOT was calculated based on each organism’s treatment indication. For organisms growing in a culture with an indication of tracheitis, any therapy beyond 5 days was considered excess (13). For organisms growing in a culture with an indication of pneumonia, any therapy beyond 7 days was considered excess (14). Calculation of excess DOT for concordant organisms helps establish a level of excess therapy that occurs outside organism reporting and may be due to individual clinician practices, stewardship impact, or patient medical history. In the overreported organism group, all DOT were considered excess DOT (Fig. 1).

### Statistical analysis.

Determination of summary statistics for all patients, cultures, and their attributable antimicrobial DOT was performed in R studio v.1.4.1106 (R Studio, Boston, MA) (Table 1 and Table 2). All regression analyses were performed using SAS statistical software v.9.4 (SAS Institute, Cary, NC). To evaluate the influence of organism reporting on antibiotic use, adjusted for clinical predictors, models were applied to the subgroup of cultures from patients with endotracheal tubes (ETTs) with isolates that were either “MURF only” or overreported, and also isolates that were either “MURF only and concordant” or overreported using reporting type as the primary predictor and DOT (postreporting and total) as the outcome (see the table labeled Supplement 4 in the supplemental material). Single predictor nested logistic regression models were used to assess the relationship between patient covariates and organism reporting (concordance), and isolate DOT (Supplement 4). Covariates with a *P* value of ≤0.10 and those that were hypothesized to be clinically relevant were selected for the final model. A negative binomial nested model was used to assess the relationship between organism reporting and either postreporting DOT or total DOT as a count outcome, using an exchangeable correlation matrix accounting for correlation of patients with multiple cultures and repeated measures and holding significant covariates constant (see Table 3 below).

**TABLE 2:** Isolate reporting and postreporting days of therapy by isolate and ventilation type

Parameter	Result for isolate group		
	Concordant	Overreported	MURF only^*a*^
Tracheostomy (*n* = 310)			
Total *n* (%)	257 (83)	25 (8)	28 (9)
Targeted for treatment, *n* (%)	95 (37)	12 (48)	6 (21)
Antibiotic started after report	47 (18)	5 (20)	3 (11)
Antibiotic started before report and continued after	48 (19)	7 (28)	3 (11)
DOT, median (IQR), range			
Postreporting	6 (3–7), 1–15 (*n* = 539)	4 (2–5), 1–9 (*n* = 48)	5 (2.5–6), 2–6 (*n* = 26)
Excess postreporting	0 (0–2), 1–10 (*n* = 115)	4 (2–5), 1–9 (*n* = 48)	0 (0–1), 0–1 (*n* = 2)
ETT (*n* = 769)			
Total *n* (%)	304 (40)	227 (29)	238 (31)
Targeted for treatment, *n* (%)	161 (53)	88 (39)	45 (19)
Antibiotic started after report	115 (38)	49 (22)	23 (10)
Antibiotic started before report and continued after	46 (15)	39 (17)	22 (9)
DOT, median (IQR), range			
Postreporting	5 (3–6), 1–13 (*n* = 809)	5 (3–7), 1–23 (*n* = 472)	4 (2–6), 1–8 (*n* = 178)
Excess postreporting	0 (0–0), 0–8 (*n* = 103)	5 (3–7), 1–23 (*n* = 472)	0 (0–0), 0–3 (*n* = 8)

a Cultures with results of “MURF only” and no other organisms identified or reported were assessed separately.

**TABLE 3:** Nested negative binomial regression models for the relationship between organism reporting and antibiotic days of therapy, accounting for control variables^*a*^

Predictor variable	*n* (%)	IRR (95% CI) for adjusted model^*b*^			
		Postreporting DOT associated with each covariate		Total DOT associated with each covariate	
		IRR (95% CI)	IRR (95% CI)	IRR (95% CI)	IRR (95% CI)
Reporting category					
Overreported vs MURF		2.83 (1.23, 6.53)*		3.24 (1.38, 7.61)*	
Overreported vs MURF and concordant			1.29 (0.75, 2.22)		1.23 (0.70, 2.15)
Age group		*P* = 0.83	*P* = 0.90	*P* = 0.89	*P* = 0.94
0–27 days	100 (22)	2.68 (0.54, 15.07)	1.30 (0.61, 2.75)	2.26 (0.42, 12.02)	1.17 (0.55, 2.50)
28 days–12 mo	130 (28)	[Reference]	[Reference]	[Reference]	[Reference]
13 mo–2 yrs	60 (13)	1.28 (0.43, 3.78)	1.07 (0.55, 2.08)	1.07 (0.35, 3.23)	0.98 (0.49, 1.95)
3–5 yrs	45 (10)	1.06 (0.30, 3.78)	1.16 (0.53, 2.54)	0.93 (0.26, 3.38)	1.16 (0.52, 2.60)
6–11 yrs	58 (12)	0.83 (0.26, 2.70)	0.85 (0.43, 1.69)	0.69 (0.21, 2.27)	0.80 (0.40, 1.61)
12–18+ yrs	71 (15)	1.22 (0.41, 3.65)	1.30 (0.65, 2.60)	1.09 (0.36, 3.34)	0.84 (0.41, 1.73)
Ventilator days at time of culture		*P* ≤ 0.01	*P* ≤ 0.01	*P* ≤ 0.01	*P* ≤ 0.01
1–7 days	221 (81)	[Reference]	[Reference]	[Reference]	[Reference]
8–30 days	35 (13)	0.05 (0.01, 0.25)*	0.18 (0.09, 0.38)*	0.06 (0.01, 0.28)*	0.20 (0.10, 0.42)*
>30 days	18 (7)	0.09 (0.01, 0.89)*	0.90 (0.39, 2.08)	0.09 (0.01, 0.94)*	0.88 (0.38, 2.06)
PMNs in Gram stain		*P* = 0.45	*P* ≤ 0.01	*P* = 0.36	*P* ≤ 0.01
Less than a few	158 (38)	0.73 (0.32, 1.64)	0.47 (0.30, 0.75)*	0.68 (0.30, 1.55)	0.41 (0.26, 0.66)*
A few or more	249 (62)	[Reference]	[Reference]	[Reference]	[Reference]
Organisms in Gram stain		*P* = 0.25	*P* = 0.16	*P* = 0.29	*P* = 0.13
No organisms seen	269 (58)	0.62 (0.28, 1.40)	0.71 (0.44, 1.14)	0.64 (0.28, 1.47)	0.69 (0.43, 1.11)
1 or more organisms seen	195 (42)	[Reference]	[Reference]	[Reference]	[Reference]

a DOT, antibiotic days of therapy; IRR, incident rate ratio; PMNs, polymorphonuclear cells. “[Reference]” represents the standard reference value.

b *, significant at *P* ≤ 0.05.

## RESULTS

Overall, 448 patients were identified for inclusion in this study (see Supplement 1 in the supplemental material). These patients had a total of 826 EACs collected, with 1,079 individual organisms reported during the study period (Supplement 1). Demographics for the patient population are reported in Table 1.

### Comparison of DOT between concordant and overreported organisms.

Comparison of postreporting DOT attributed to concordant, MURF-only, and overreported organisms are presented in Table 2. Of the reported organisms from tracheostomy cultures (*n* = 310), 257 (83%) were reported in concordance with ASM guidelines, 25 (8%) were overreported, and 28 (9%) were MURF only (Table 2). Of reported organisms from tracheostomy cultures, targeted treatment and initiation of an antibiotic were most common in the overreported group (48%). The postreporting DOT associated with the targeted treatment of concordant organisms from cultures in the tracheostomy group was 539 (median, 6; interquartile range [IQR], 3, 7). Of these, 115 days were considered excess DOT (median, 0; IQR, 0, 2). The overreported organism group contributed 48 excess postreporting DOT overall (median, 4; IQR, 2, 5) (Table 2). The MURF-only isolates contributed 26 postreporting DOT, 2 of which were considered excess DOT.

Among isolates reported from the cultures of patients with ETTs (*n* = 769), 29% of organisms were overreported (*n* = 227), 39% were concordant (*n* = 304), and 31% (*n* = 238) were MURF only. Of organisms reported from ETT cultures, targeted treatment was most common in the concordant group (53%). The postreporting DOT associated with the targeted treatment of concordant organisms in the ETT group was 809 days (median, 5; IQR, 3, 6). Of these, 103 days were considered excess DOT (median, 0; IQR, 0, 0). In the overreported group, 88 (39%) organisms were treated and treatment was started after organism reporting in 56% of isolates. This group of organisms contributed 472 excess postreporting DOT overall (median, 5; IQR, 3, 7) (Table 2). In the MURF-only group, there were 178 postreporting DOT (median, 4; IQR, 4, 6). Overall, 8 of those postreporting DOT were considered excess (Table 2).

### Organisms overreported from ETT specimens.

**FIG 2:**
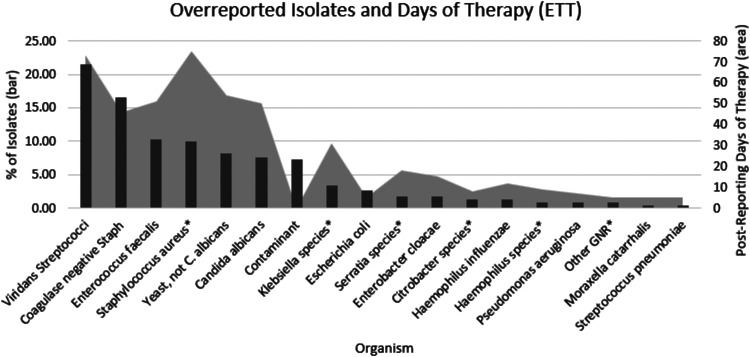
Overreported isolates, day of therapy, and endotracheal tube (ETT) specimen cultures. “Staphylococcus aureus*” represents methicillin-resistant *S. aureus*, methicillin-susceptible *S. aureus*, and *S. aureus* not further identified. “Contaminant” represents *Abiotrophia* species, Bacillus cereus group, coryneform bacteria, Gram-positive organism not identified, nonpathogenic *Neisseria* species, and *Rothia* species. “Klebsiella species*” represents Klebsiella oxytoca and Klebsiella pneumoniae. “Serratia species*” represents Serratia marcescens and *Serratia* species not further identified. “Citrobacter species*” represents Citrobacter koseri and Citrobacter freundii. “Other GNR*” represents *Aeromonas* species and *Pseudomonas* species not *P. aeruginosa*. “Haemophilus species*” represents *H. haemolyticus*.

The most commonly overreported organisms in the ETT group were viridans group streptococci (22%), coagulase-negative staphylococci (CoNS) (17%), Enterococcus faecalis (10%), Staphylococcus aureus (10%), Candida albicans (8%), and yeast not Candida albicans (8%) (Fig. 2). S. aureus (75 postreporting DOT), viridans group streptococci (73 postreporting DOT), Yeast not C. albicans (54 postreporting DOT), E. faecalis (51 postreporting DOT), C. albicans (50 postreporting DOT), and CoNS (46 postreporting DOT) were associated with the most postreporting DOT (Fig. 2). Amoxicillin-clavulanate, ceftriaxone, cefepime, ampicillin-sulbactam and amoxicillin were the most prescribed antibiotics (see the figure labeled Supplement 2 in the supplemental material).

### Analysis of the impact of overreported organisms versus MURF only.

Covariates were selected *a priori* for single-variable analysis and included age, gender, race, presence of underlying medical conditions, length of stay at time of culture, cumulative ventilator days at time of culture, PMNs in Gram stain, and organisms in Gram stain (see Supplement 4 in the supplemental material). Several covariates were associated with organism reporting. In the first model comparing overreported isolates to MURF, the 0- to 27-day age group was significantly more likely to have an overreported isolate compared to older children (odds ratio [OR], 10.36; 95% confidence interval [CI], 2.99, 35.81), and patients without an underlying condition at the time of admission had four times the odds of having an overreported organism compared to patients with one or more underlying conditions prior to admission (OR, 4.26; 95% CI, 1.82, 9.92). Length of stay and number of ventilator days at the time of culture collection were both significantly associated with organism reporting (*P* < 0.05), while PMNs and organisms seen in the Gram stain were not significantly associated with overreporting (Supplement 4). Significant relationships were seen between organism reporting and the variables age group, underlying medical conditions, length of stay, and ventilator days using the second model (overreported versus MURF and concordant). Unlike the first model, lack of PMNs and organisms seen in the original Gram stain were significantly associated with organism overreporting (*P* < 0.05).

Ventilator days at the time of culture collection was a parameter significantly associated with postreporting DOT and total DOT (*P* < 0.05), and isolates in cultures with an original negative Gram stain were associated with significantly less postreporting DOT (incident rate ratio [IRR], 0.57; 95% CI, 0.36, 0.90) and less total DOT (IRR, 0.51; 95% CI, 0.32, 0.81) than isolates from cultures with a positive Gram stain. Ultimately, age group, ventilator days, PMNs, and organisms in Gram stain were selected for entry into the final model (Supplement 4).

After adjustment, organism overreporting was associated with a nearly 3-fold-higher rate of postreporting DOT (IRR, 2.83; 95% CI, 1.23, 6.53) and >3-fold-higher rate of total DOT (IRR, 3.24; 95% CI, 1.38, 7.61) compared to MURF-only reporting. When all concordant organisms were considered (MURF and individual concordant organisms), there was not a significant difference between overreported and concordant organisms with respect to postreporting DOT or total DOT (*P* > 0.05) (Table 3).

## DISCUSSION

In this single-center retrospective study, we found that microbiology overreporting of organisms from EACs was associated with excess antimicrobial exposure in mechanically ventilated patients. This study design is unique in that data were collected at the organism level and focused on the influence of microbiology practices on clinician decision-making (to target the organism for therapy) and patient antibiotic exposure. Previous studies demonstrate that higher rates of EAC use in hospitals are associated with higher rates of antimicrobial use (1). While the reason for this is likely multifaceted, this study supports the hypothesis that laboratory reporting practices for EACs influence prescribing choices.

ASM guidelines recommend that organisms that are not known lower respiratory pathogens or organisms that may indicate colonization should be reported as normal respiratory flora. The principle behind this is that fully identifying and reporting organisms from poorly collected samples or nonsterile sites may prompt unnecessary antimicrobial use. While individualized decision-making may be warranted in some cases, clinical decision-making around complex and frequently contaminated specimens such as urine and EACs has been shown to be variable and unreliable (15). A critical role of the clinical microbiology lab is to support antimicrobial stewardship through results reporting, which often includes the framing of culture results to avoid unnecessary antimicrobial use (16, 17).

In contrast to patients with tracheostomies, 29% of organisms reported from the EACs of patients with ETTs were overreported, which contributed to a significant number of excess DOT. Although it is not possible to know if the patient would have been treated if the organism had not been fully identified and reported, the clinically documented targeted initiation and continuation of therapy based on the culture report suggest that the reported organism was at least partially responsible for the initiation of therapy. It is possible that when contaminants or colonizing organisms are fully identified and reported from EACs—notably when other tests do not support clinical symptoms—these are interpreted as the only apparent cause and are treated as such.

The findings from this study demonstrate that reporting of EAC isolates by the microbiology laboratory may influence clinician antimicrobial prescribing behavior. It is important to note that clinicians overtreat even in the absence of an overreported organism, as demonstrated by the excess DOT in the concordantly reported group. However, this study highlights the importance of targeting organism reporting practices to decrease excess DOT associated with respiratory cultures.

While previous studies focus primarily on studying and modifying behavior of clinical practitioners (18), this study identifies possible target areas for stewardship interventions in the microbiology laboratory. Findings from this study suggest that modifying EAC organism reporting procedures during the post-analytical phase in the laboratory to align with accepted reporting guidelines could significantly reduce the number of excess DOT ventilated patients receive.

### Limitations.

This study has limitations. First, a significant challenge of this type of study is that prescribing behavior is difficult to assess retrospectively and can differ between and within hospital units. To address this, we executed a manual chart review process that searched for explicit language describing the targeted treatment of a reported organism or antibiotic indications specifically indicating pneumonia or tracheitis with an antibiotic generally accepted as used to treat the reported organism. This methodology greatly improved targeted DOT data accuracy, although since clinical signs of pneumonia were not considered, there may still be some DOT that are misclassified. While longer durations of treatment outside 5 or 7 days may be appropriate for certain organisms (i.e., Pseudomonas aeruginosa) and the organism identification was not considered when calculating excess DOT, it is possible that the values for excess DOT are slightly overestimated. However, nonfermenting Gram-negative rods comprised a small proportion of the analyzed cohort, and the impact of this potential misclassification is likely small. Due to the depth of the data collected, culture and DOT data were only collected from each patient’s first admission during the study period. Only the organism identification that was initially reported by the microbiology laboratory was included. If a provider called the laboratory and requested additional workup, this may have impacted antibiotic choice and duration and was not assessed in final analyses. The clinical indication (by LRTI diagnosis or other) was not independently assessed when classifying excess DOT, but this is addressed by considering total DOT in the modified analysis. DOT data collection was limited to three antibiotics for each isolate. While the use of more than three antibiotics for each isolate is unlikely, this is a possible limitation of the study design.

Finally, this is a single-center study, and the findings may not be generalizable to other institutions, particularly given the variability in EAC processing and reporting (8). To address this, we used ASM guidelines to determine the concordance of organism reporting. While these guidelines are the best available standard for organism reporting from lower respiratory culture, their interpretation and use are variable. Reporting practices vary by laboratory setting, clinician needs and preferences, and patient population (8). We performed a culture result adjudication process between two microbiologists to ensure that the guidelines were being applied to culture results in a similar way, but this may have limited generalizability because both microbiologists trained at the same institution.

### Conclusions.

Organism overreporting from EACs impacts antimicrobial prescribing behavior and contributes to excess therapy in mechanically ventilated patients. Further studies are needed that demonstrate the impact of interventions that modify culture reporting practices in the laboratory and the impact on key clinical outcomes.
