# Supplementary material for: Impact of Organism Reporting from Endotracheal Aspirate Cultures on Antimicrobial Prescribing Practices in Mechanically Ventilated Pediatric Patients
Source: J Clin Microbiol. 2022 Oct 11;60(11):e00930-22. doi: 10.1128/jcm.00930-22 (PMC9667758; doi:10.1128/jcm.00930-22)
Supplement: Supplemental file 1 — Supplemental material. Download jcm.00930-22-s0001.pdf, PDF file, 0.5 MB [file jcm.00930-22-s0001.pdf]

Supplement 1. Inclusion and Exclusion of Patients and Cultures

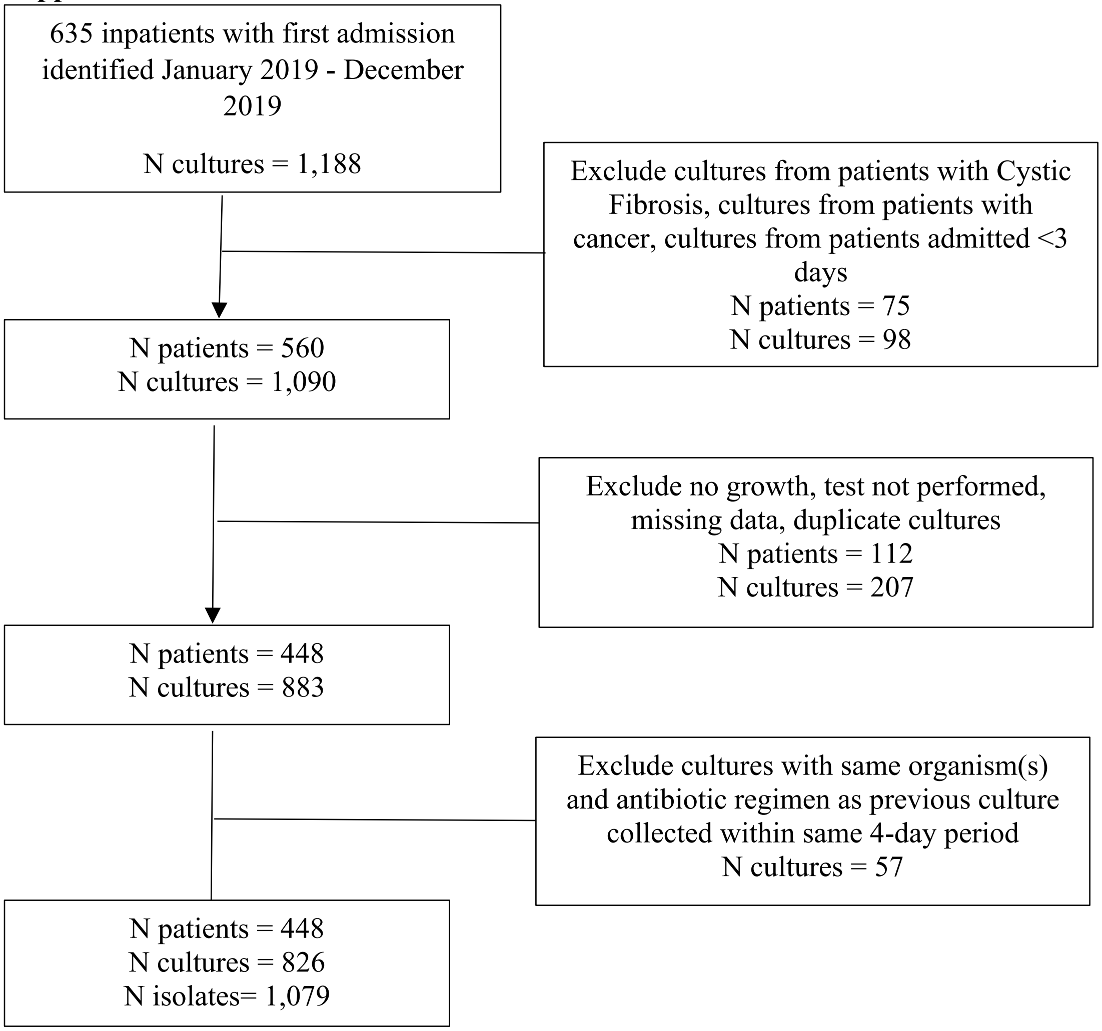

*EAC = Endotracheal aspirate culture

Supplement 2. Antibiotics Prescribed and Associated Days of Therapy

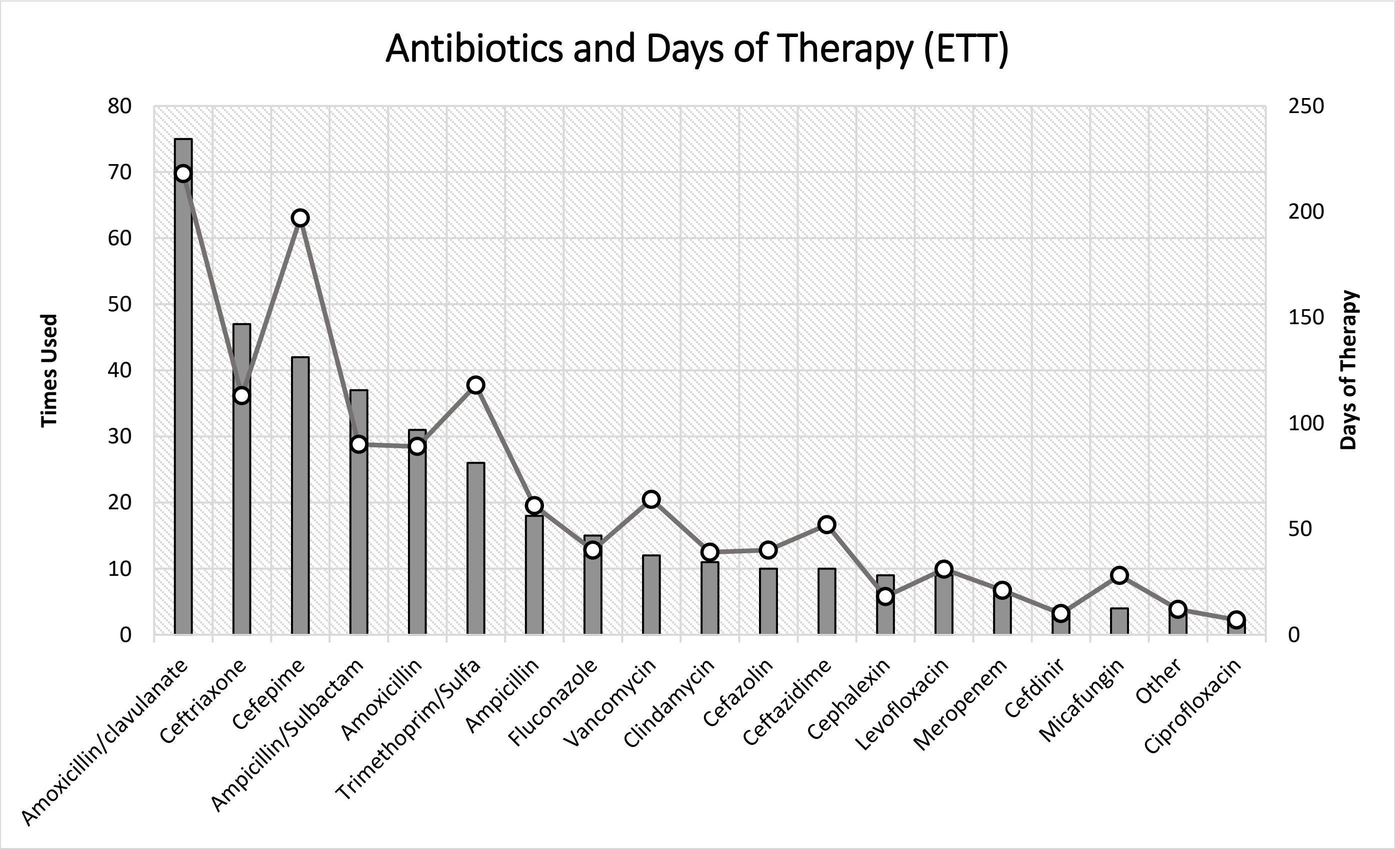

Supplement 3. Organism Classification Flowcharts

Always report the following, regardless of quantity or predominance:

1. Group A strep
2. Group B Strep (pediatrics)
3. *Francisella tularensis*
4. *Bordetella species*
5. *Yersinia pestis*
6. GC
7. *Bacillus anthracis*
8. *Cryptococcus neoformans*
9. Mold

The following are ALWAYS mixed upper respiratory flora (MURF) and should never be reported individually. If any of these are reported in culture, that is considered overreporting:

1. Viridans Strep
2. Non pathogenic *Neisseria*
3. Diphtheroids
4. Coag neg staph
5. *Rothia*
6. Group F strep (*Strep anginosus*)
7. Anaerobes
8. *Haemophilus* species not influenzae
9. *Eikenella*
10. *Acintobacillus*
11. *Capnocytophaga*
12. *Moraxella* species not catarrhalis
13. Enterococci
14. Yeast
15. *Abiotrophia*
16. *Bacillus cereus* group

17. *Lactobacillus*
18. *Pasteurella*

Supplement 3. Organism Classification Flowcharts

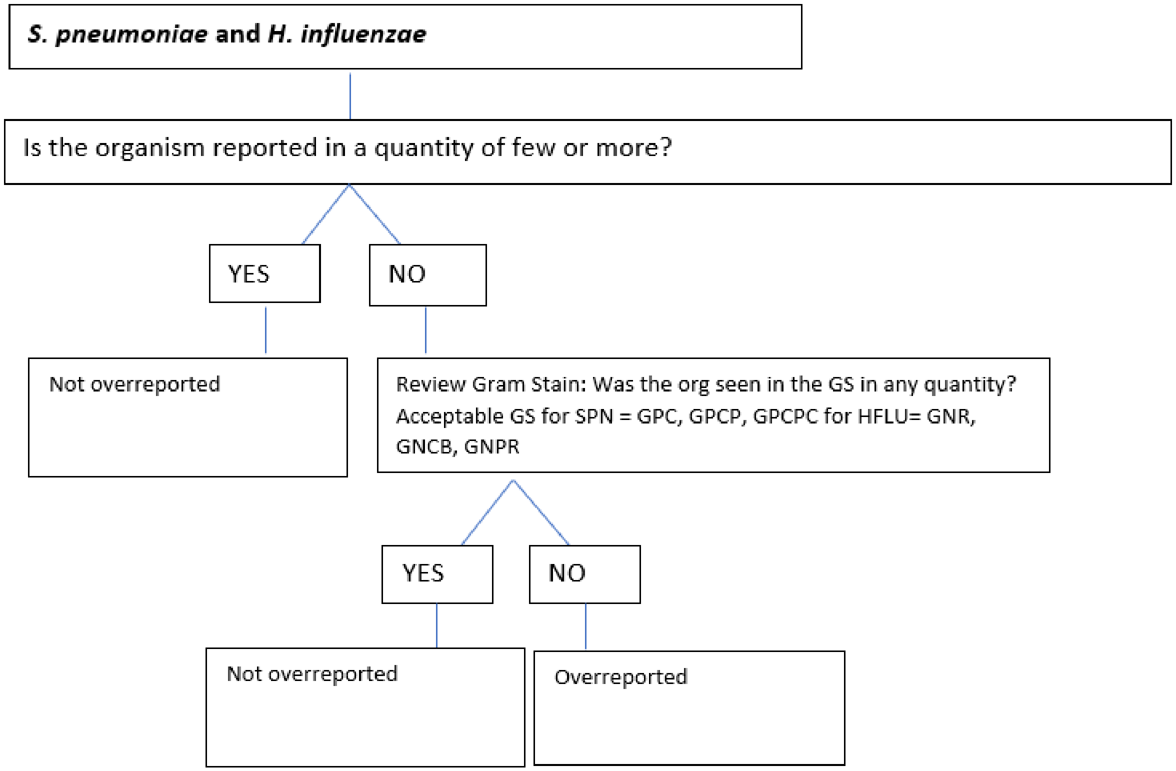

Supplement 3. Organism Classification Flowcharts

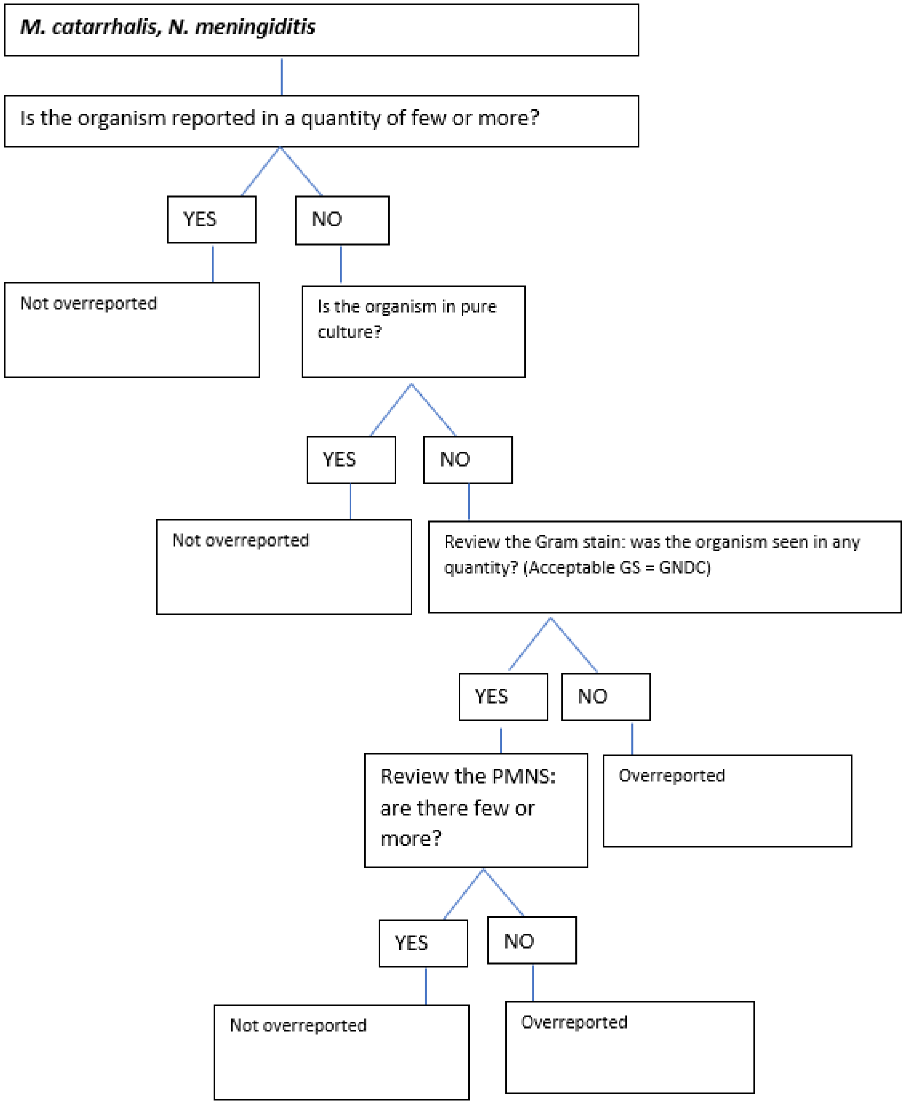

Supplement 3. Organism Classification Flowcharts

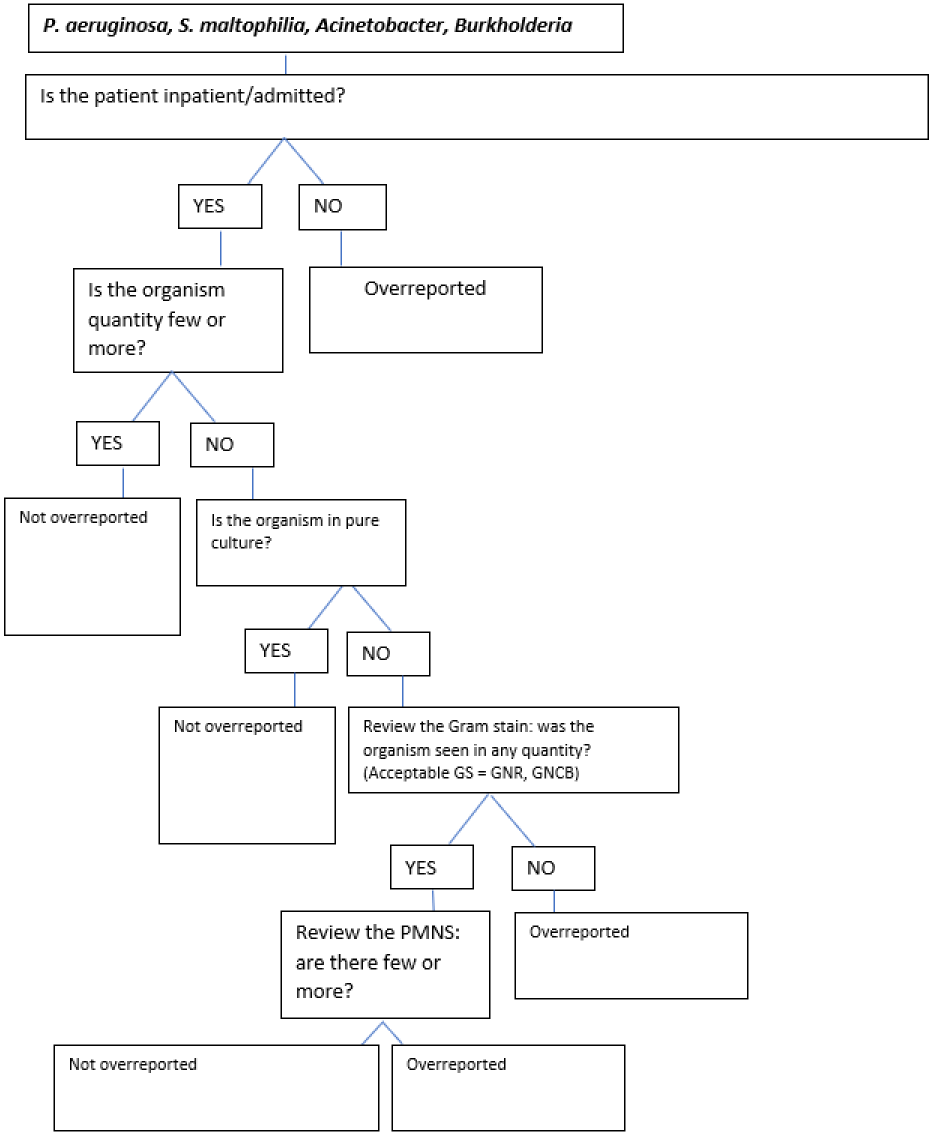

Supplement 3. Organism Classification Flowcharts

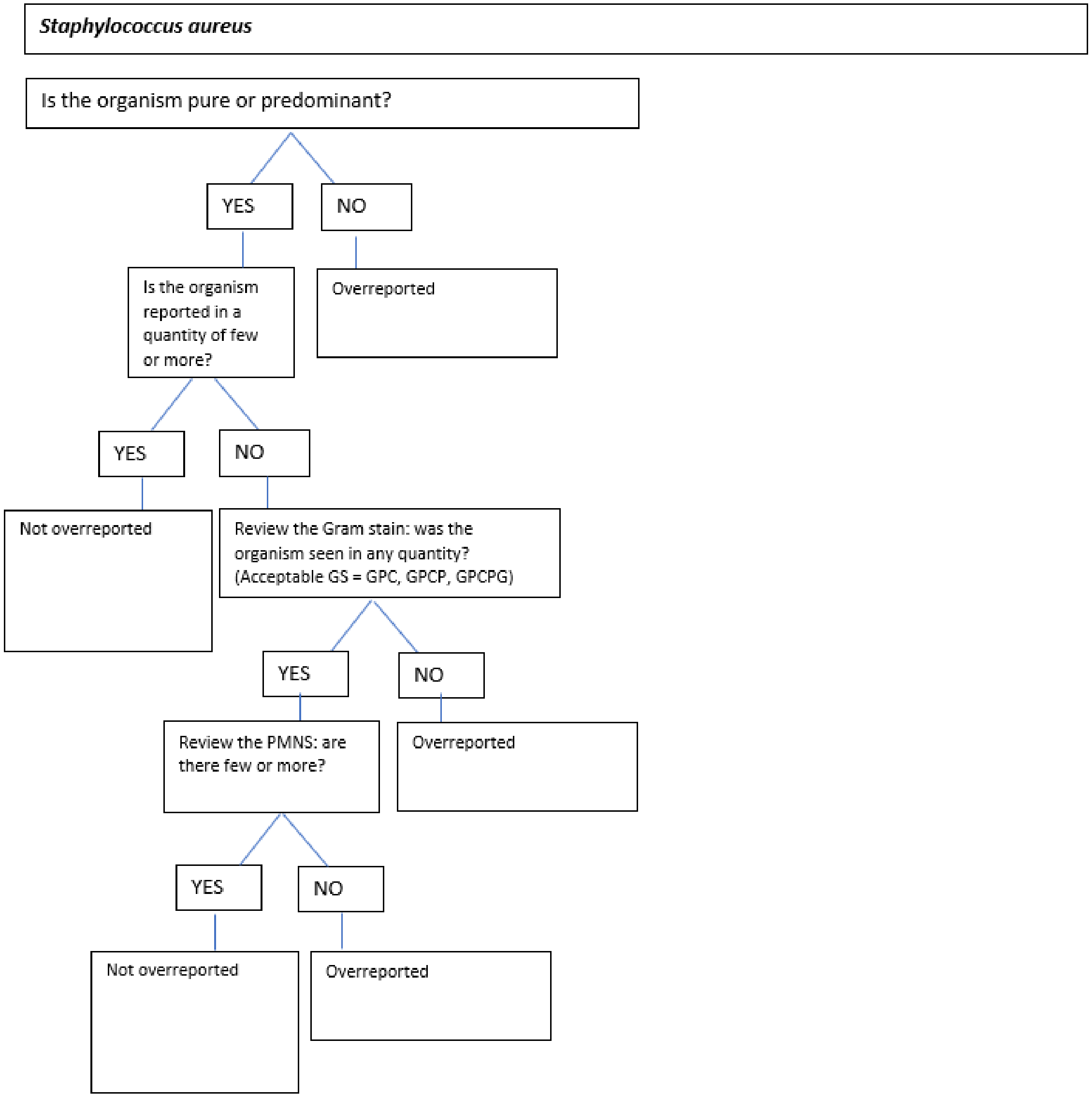

Supplement 3. Organism Classification Flowcharts

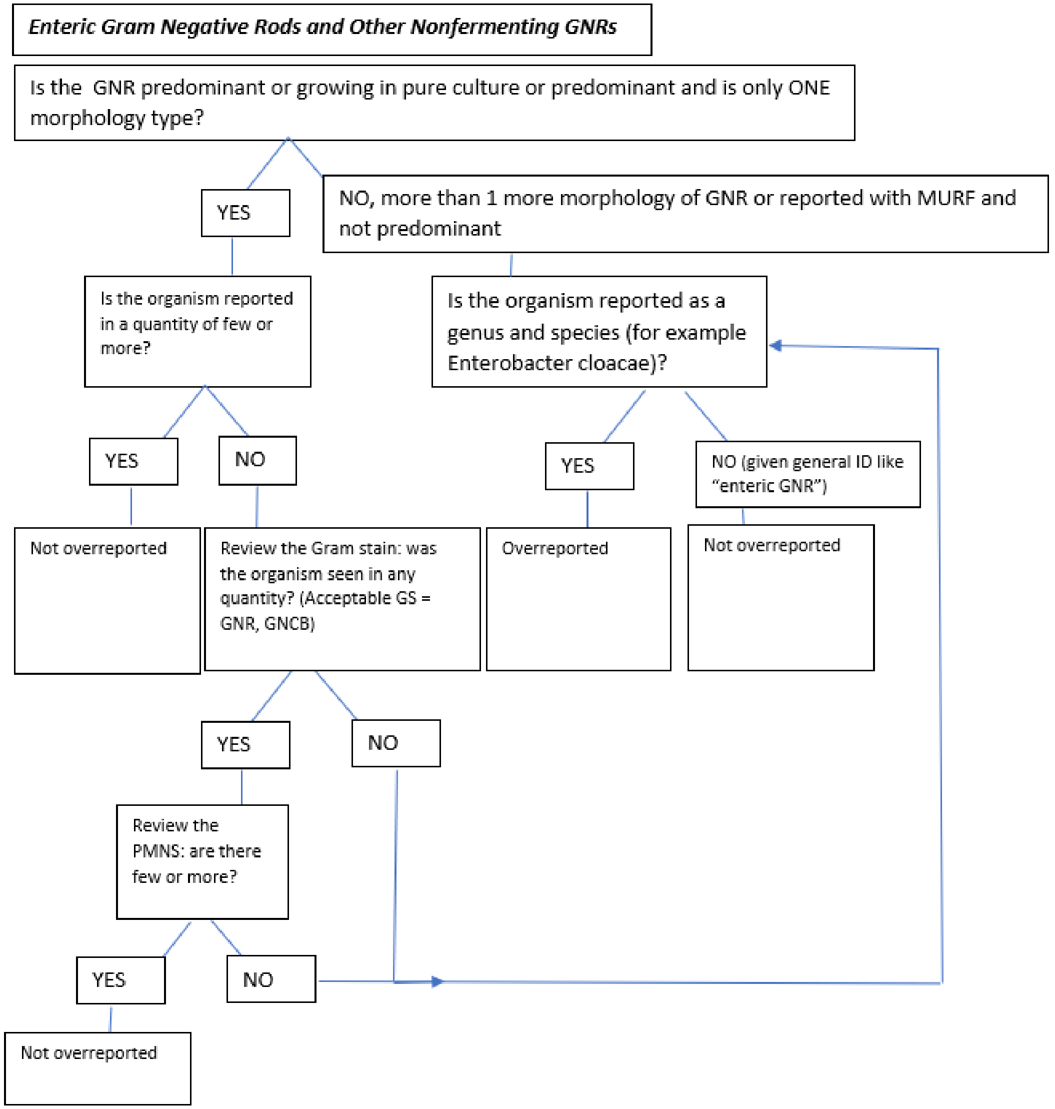

Supplement 3. Organism Classification Flowcharts

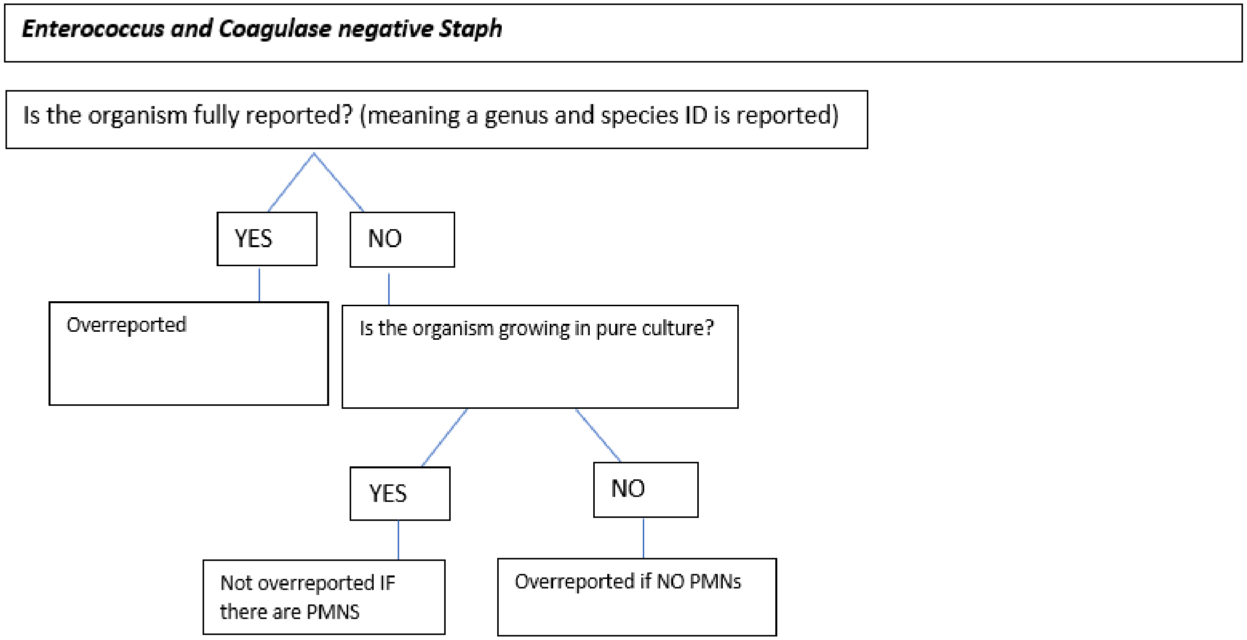

Supplement 4. Single Predictor Nested Logistic Regression Models Predicting Report Category and Days of Therapy (DOT)

Variable	N (%)	OR (95% CI) Bivariate: Report category (Overreported vs MURF)	OR (95% CI) Bivariate: Report Category (Overreported vs MURF & Concordant)	IRR (95%CI) Bivariate: Post-reporting Days of Therapy	IRR (95% CI) Bivariate: Total Days of Therapy
**Age Group**		**p≤0.01**	**p≤0.01**	p=0.08	**p=0.03**
0-27 days	100 (22)	**10.36 (2.99, 35.81) ***	**5.17 (1.81, 14.80) ***	1.36 (0.82, 2.26)	1.40 (0.89, 2.50)
28 days – 12 months	130 (28)	[Reference]	[Reference]	[Reference]	[Reference]
13 months-2 years	60 (13)	**0.09 (0.02, 0.39) ***	**0.20 (0.05, 0.72) ***	0.72 (0.37, 1.39)	0.71 (0.36, 1.40)
3-5 years	45 (10)	0.52 (0.13, 2.12)	1.30 (0.33, 4.89)	0.84 (0.39, 1.82)	0.91 (0.42, 2.00)
6-11 years	58 (12)	0.32 (0.08, 1.30)	0.50 (0.13, 1.80)	0.51 (0.25, 1.04)	0.23 (0.25, 1.08)
12-18+ years	71 (15)	0.52 (0.15, 1.73)	1.18 (0.38, 3.72)	0.69 (0.35, 1.35)	0.59 (0.29, 1.19)
**Gender**		p=0.75	p=0.38	p=0.50	P=0.77
Female	207 (45)	1.14 (0.51, 2.55)	1.39 (0.66, 2.89)	0.86 (0.56, 1.34)	0.94 (0.60, 1.46)
Male	257 (55)	[Reference]	[Reference]	[Reference]	[Reference]
**Race**		**p=0.01**	**p=0.03**	p=0.70	p=0.84
Black, not Hispanic or Latino	35 (7)	0.24 (0.05, 1.08)	0.45 (0.10, 1.96)	1.56 (0.68, 3.61)	1.32 (0.57, 3.10)
Hispanic or Latino	135 (29)	1.10 (0.44, 2.72)	0.80 (0.35, 1.83)	0.94 (0.57, 1.56)	0.92 (0.55, 1.52)
Other	92 (20)	**0.20 (0.07, 0.61) ***	**0.22 (0.08, 0.62) ***	1.02 (0.68, 3.61)	1.07 (0.58, 2.00)
White, not Hispanic or Latino	202 (44)	[Reference]	[Reference]	[Reference]	[Reference]
**Medical History**		**p≤0.01**	**p=0.02**	p=0.36	p=0.38
One or more complex conditions at Admission	272 (59)	[Reference]	[Reference]	[Reference]	[Reference]
No complex conditions at admission	192 (41)	**4.26 (1.82, 9.92) ***	**2.42 (1.13, 5.23) ***	1.21 (0.80, 1.81)	1.25 (0.76, 2.07)
**Length of stay at time of culture (days)**		**p≤0.01**	p=0.09	p=0.33	p=0.44
Less than 1 day	69 (17)	0.39 (0.13, 1.13)	0.35 (0.13, 0.98)	1.36 (0.72, 2.58)	1.35 (0.70, 2.61)
1-3 days	144 (36)	[Reference]	[Reference]	[Reference]	[Reference]
4-10 days	76 (19)	1.69 (0.67, 4.24)	1.85 (0.76, 4.49)	1.09 (0.59, 2.02)	1.04 (0.55, 1.98)
11-30 days	51 (13)	**4.47 (1.45, 13.80) ***	1.48 (0.57, 3.82)	1.95 (1.03, 3.71)	1.81 (0.93, 3.53)
>30 days	59 (15)	**3.78 (1.22, 11.70) ***	1.63 (0.60, 4.43)	1.25 (0.70, 3.71)	1.36 (0.75, 2.47)
**Ventilator days at time of culture**		**p=0.04**	p=0.27	**p≤0.01**	**p≤0.01**
1-7 days	221 (81)	[Reference]	[Reference]	[Reference]	[Reference]
8-30 days	35 (13)	**3.84 (1.25, 11.80) ***	1.90 (0.74, 4.89)	**0.21 (0.11, 0.41) ***	**0.22, (0.11, 0.43) ***

>30 days	18 (7)	2.74 (0.60, 13.02)	0.75 (0.23, 2.40)	1.05 (0.52, 2.12)	1.04 (0.51, 2.14)
**Polymorphonuclear cells (PMNs) in Gram Stain**		p=0.31	**p=0.03**	p=0.09	**p=0.02**
Less than few	158 (38)	1.40 (0.73, 2.66)	**1.87 (1.06, 3.28) ***	0.62 (0.36, 1.10)	**0.53 (0.32, 1.10) ***
Few or more	249 (62)	[Reference]	[Reference]	[Reference]	[Reference]
**Organisms in Gram Stain**		p=0.11	**p=0.04**	**p=0.02**	**p≤0.01**
No organisms seen	269 (58)	0.59 (0.31, 1.12)	**1.73 (1.01, 2.95) ***	**0.57 (0.36, 0.90) ***	**0.51 (0.32, 0.81) ***
One or more organisms seen	195 (42)	[Reference]	[Reference]	[Reference]	[Reference]

*Significant at a p value of ≤0.05

Abbreviations: OR = odds ratio, IRR = Incident rate ratio
